# Determination of flutamide and two major metabolites using HPLC–DAD and HPTLC methods

**DOI:** 10.1186/s13065-018-0372-y

**Published:** 2018-01-25

**Authors:** Nada S. Abdelwahab, Heba A. H. Elshemy, Nehal F. Farid

**Affiliations:** 1 Pharmaceutical Analytical Chemistry, Faculty of Pharmacy, Beni-suef University, Beni-Suef, Egypt; 2 Pharmaceutical Chemistry, Faculty of Pharmacy, Nahda University (NUB), Sharq El-Nile, Beni-Suef, 62511 Egypt; 3 Pharmaceutical Organic Chemistry, Faculty of Pharmacy, Beni-Suef University, Beni-Suef, Egypt

**Keywords:** Flutamide, Metabolites, HPTLC, HPLC, Plasma, Urine

## Abstract

Flutamide is a potential antineoplastic drug classified as an anti-androgen. It is a therapy for men with advanced prostate cancer, administered orally after which it undergoes extensively first pass metabolism in the liver with the production of several metabolites. These metabolites are predominantly excreted in urine. One of the important metabolites in plasma is 4-nitro-3-(trifluoromethyl)phenylamine (Flu-1), while the main metabolite in urine is 2-amino-5-nitro-4-(trifluoromethyl)phenol (Flu-3). In this work the two metabolites, Flu-1 and Flu-3, have been synthesized, and then structural confirmation has been carried out by HNMR analysis. Efforts were exerted to develop chromatographic methods for resolving Flutamide and its metabolites with the use of acceptable solvents without affecting the efficiency of the methods. The drug along with its metabolites were quantitatively analyzed in pure form, human urine, and plasma samples using two chromatographic methods, HPTLC and HPLC–DAD methods. FDA guidelines for bio-analytical method validation were followed and USP recommendations were used for analytical method validation. Interference from excipients has been tested by application of the methods to pharmaceutical tablets. No significant difference was found between the proposed methods and the official one when they were statistically compared at p value of 0.05%.

## Introduction

Flutamide has chemical structure of 2-methyl-N[4-nitro-3-(trifluoromethyl)phenyl]propanamide [1]. It is an acetanilide, non-steroidal orally active anti-androgen [2] used clinically for the management of metastatic carcinoma [3]. Patients treated with Flutamide developed severe hepatotoxicity that is thought to be as a result of its toxic metabolites [4]. Metabolism of Flutamide occurs by human liver microsomes after 1 h from oral administration with the production of many toxic metabolites. 4-nitro-3-(trifluoromethyl)phenylamine [Flu-1] is reported to be one of the important Flutamide plasma metabolites [5] and also one of its impurities and related substances according to BP [6] and USP [7]. Flu-1 is proved to cause severe hepatic dysfunction [5] and is found to be the major hydrolytic degradation product of the anticancer Flutamide [8]. On the other hand, 2-amino-5-nitro-4-(trifluoromethyl)phenol (Flu-3) is an inactive metabolite and the main one in urine that represents from 50 to 90% of urinary excretion [4].

Flutamide is a pharmacopoeial drug reported in BP [6] and USP [7]. In BP [6] Flutamide was determined by a spectrophotometric method, while in USP [7] it was measured in both pure form and capsules by a RP-HPLC method using C18 column.

Other methods were published for determination of Flutamide including electrochemical [2, 9, 10], different spectrophotometric [2, 8, 11–14], spectrofluorimetric [15], and different chromatographic methods [2, 3, 16–20].

Solvents in any developed analytical method are of great importance, most solvents are organic with hazardous and toxic properties causing environmental and health problems [21]. Chromatographic methods are widely used for qualitative and quantitative analysis. It is used for resolving complex mixtures [22], during stability studies [23], determination of drugs and their impurities [24], and determination of drugs in biological fluids [24].

Synthesis of the metabolites has been successfully carried out in our laboratory and structural confirmation has been performed. In addition, in this work we were concerned with the development and validation of two highly sensitive and selective chromatographic methods, HPTLC and HPLC–DAD methods, using developing systems with the least hazardous solvents and the maximum chromatographic resolution. The developed methods were applied for determination of Flutamide in raw material and marketed tablets. Moreover, application of the methods was extended for determination of the drug and its metabolites in human plasma and urine samples. The developed HPTLC method is the first one reported for separation and quantitation of Flutamide and its metabolites, while the HPLC–DAD method has high selectivity, precision, and short analysis time (< 10 min). Moreover, the developed methods have advantages of lower cost comparing to previously reported LC–MS methods [4, 5]. Additionally, the facilities required for the methods developed in this article are mostly available in all laboratories, allowing them to be commonly applied for drug monitoring. The methods developed below are the only ones concerned with quantification of the drug along with its metabolites.

## Experimental

### Instruments

#### For HPTLC method

Samples were applied by CAMAG Linomat 5, auto-sampler (Switzerland) using CAMAG micro-syringe, 100 µL (Switzerland) on HPTLC aluminum plates, pre-coated with silica gel 60 F254 (20 × 20 cm) (Merck, Germany), 200 µm thickness and 5 µm particle size. Chromatographic development was performed in glass chamber (Macherey–Nagel, Germany). In the initial trials and during method optimization, detection of the drug and the metabolites was done using UV Lamp-short wavelength 254 nm. Finally, scanning was carried out using CAMAG TLC densitometric Scanner 3S/N 130319 with WINCATS software (CAMAG, Muttens, Switzerland).

#### For HPLC method

Chromatographic separation was carried out on HPLC instrument (Agilent 1260 Infinity, Germany) equipped with a G1361A pump, G1316A thermo-stated column compartment, and G2260A auto-sampler. The detector used was G131SD diode array detector VL, while the stationary phase was ZORBAX Eclipse Plus CN column (150 × 4.6 mm i.d, 5 µm particle size) (USA).

### Materials

#### Pure samples

Flutamide (Sigma-Aldrich chemie GmbH., Germany) with a purity of 99.25% according to the official method [6].

#### Pharmaceutical formulation

Cytomed-250^®^ tablets, was manufactured by CIPLA LTD. INDIA. It was labeled to contain 250 mg Flutamide per tablet.

#### Biological samples

Blank human plasma and urine samples were supplied by Dr./Khaled Nagy Laboratory, Beni-suef, Egypt and they were obtained from healthy volunteers.

### Chemicals and reagents

#### For synthesis

Methanol, chloroform, HCl, glacial acetic acid, dichloromethane, iodine mono chloride, sodium bicarbonate, sodium hydroxide, and magnesium sulphate (El-Nasr Pharmaceutical Chemicals Co., Abu-Zabaal, Cairo, Egypt).

#### For analysis

Toluene (El-Nasr Pharmaceutical Chemicals Co., Abu-Zabaal, Cairo, Egypt).

Tetrahydrofuran, methanol, and acetonitrile (HPLC grade, [(Tedia, USA), (Fisher Scientific, UK)].

Deionized water (SEDICO Pharmaceuticals Co., Cairo, Egypt).

#### Solutions

##### Stock solutions of Flutamide, Flu-1 and Flu-3: (1 mg/mL)

They were prepared by accurately weighing 0.1 gm of each in three separate 100 mL volumetric flasks and dissolving in either methanol (for HPTLC) or acetonitrile (for HPLC–DAD).

##### Working solutions of Flutamide, Flu-1 (0.2 mg/mL) and Flu-3 (0.5 mg/mL) [for HPLC–DAD]

They were prepared by transferring either 20 mL (for Flutamide and Flu-1) or 50 mL (for Flu-3) from their respective stock solutions (1 mg/mL) into three separate 100 mL calibrated flasks, the volume of each flask was completed with the mobile phase, acetonitrile–water (40:60, v/v).

#### Synthesis of flutamide metabolites

##### Synthesis of 4-nitro-3-(trifluoromethyl)phenylamine [Flu-1]

Method developed by Farid and Abdelwahab [8] has been followed during preparation of Flu-1.

##### Synthesis of 2-amino-5-nitro-4-(trifluoromethyl)phenol (Flu-3)

**Fig. 1:**
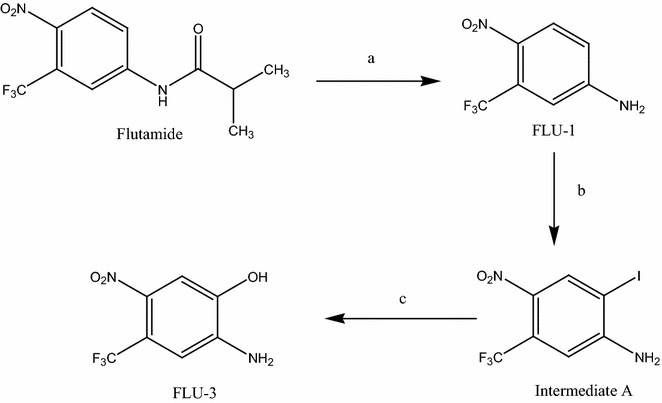
Scheme for preparation of Flu-3. Reagents and conditions: (a) NaOH, methanol, reflux, 3 h, (b) ICl, acetic acid, RT, 1.5 h; (c) aqueous NaOH, reflux, 24 h

Synthesis of Flu-3 was carried out according to the synthetic pathway depicted in Fig. 1.

###### General method for preparation of 2-iodo-4-nitro-5-trifluoromethyl-phenylamine (Intermediate A)

A solution of iodine monochloride (0.017 M) in glacial acetic acid (35 mL) was added drop wise over 10 min at 25 °C to a solution of Flu-1 (0.013 M) in glacial acetic acid (35 mL). The mixture was stirred at 25 °C for a further 1.5 h and excess glacial acetic acid was then removed by vacuum evaporation. The residue was partitioned between aqueous sodium bicarbonate-dichloromethane and the separated organic layer was washed with water (2 × 60 mL), dried (MgSO_4_), and re-crystallized from methanol to afford intermediate A.

###### General method for preparation of 2-amino-5-nitro-4-trifluoromethyl-phenol (Flu-3)

A solution of intermediate A (0.01 M) in aqueous sodium hydroxide solution 15% (25 mL) was heated under reflux for 24 h. After cooling, the reaction mixture was acidified with hydrochloric acid and the formed solid was filtered, washed with water, dried and re-crystallized from methanol: chloroform (1:1) to afford Flu-3.

### Pharmaceutical formulation sample

Ten cytomed-250^®^ tablets were grinded and then accurately weighed. An amount of the powdered tablets equivalent to 200 mg Flutamide was transferred into 100 mL volumetric flask, 75 mL of either methanol (for HPTLC) or acetonitrile (for HPLC–DAD) was added and the solution was ultra-sonicated for 30 min. The solution was filtered and then the appropriate solvent was added till adjusting the volume to prepare sample stock solution of (2 mg/mL). Working solution (0.2 mg/mL) *[for HPLC*–*DAD]* was then prepared in the mobile phase mixture of acetonitrile–water (40:60, v/v).

### Procedure

#### Linearity

##### Pure samples

###### For HPTLC

Different concentrations of Flutamide, Flu-1, and Flu-3 in the range of 10–350 µg/mL were prepared in methanol from their corresponding stock solutions. 10 µL were applied in triplicates from each concentration to HPTLC plates. They were applied as bands of 6 mm width using a micro-syringe, the bands were spaced by a distance of 8.9 mm. Scanning speed was set at 20 mm/s and the slit dimension was adjusted to 6.0 × 0.3 µm. A glass chamber saturated with the mobile phase consisting of toluene: tetrahydrofuran: glacial acetic acid (8:2:0.2, by volume) for half an hour was prepared and the chromatographic development was left until the mobile phase migrated to 8 cm. UV scanning was done at 370 nm. The results were recorded as peak areas which together with the corresponding concentrations were then used to calculate the regression equations of each component.

###### For HPLC

Different samples of Flutamide, Flu-1, and Flu-3 were prepared from their respective working solutions in the concentration ranges of 2–50, 1–50, and 5–200 µg/mL for Flutamide, Flu-1 and Flu-3, respectively. Separation was done on CN column using a mobile phase consisting of acetonitrile: water (40:60, v/v) with a flow rate of 1 mL/min at ambient temperature. The detector was adjusted at 220, the injection volume was 20 µL and the run time was adjusted at 10 min. The peak areas were recorded and used for construction of their calibration curves.

##### Spiked human plasma samples

###### For HPTLC method

Into three separate sets of 5 mL volumetric flasks, different concentrations of Flutamide, Flu-1, and Flu-3 samples in the range of 30–300 µg/mL were prepared, 0.5 mL plasma was added to each flask and 1 mL methanol was then used to precipitate plasma protein. The volume was completed with methanol.

###### For HPLC method

Samples in the range of 2–50 µg/mL for both Flutamide and Flu-1 and in the range of 15–200 µg/mL for Flu-3 were separately transferred from their previously prepared working solutions into three separate sets of 5 mL volumetric flasks. 0.5 mL plasma was added to each flask, then 1 mL acetonitrile was added to precipitate the plasma protein and volume was then completed with the mobile phase.

The prepared solutions were then vortexed for 1 min. To remove the precipitated plasma protein, samples were placed in a cooling centrifuge for 5 min at 5000 rpm and then samples were filtered through 0.45 μm rated Acrodisc MS syringe filter (PN MS-3201). Procedure under linearity for each method has been followed, peak areas were then recorded, and regression equations have been computed.

##### Spiked human urine samples

###### For both HPTLC and HPLC methods

Solutions of different concentrations in the range of 30–400 µg/mL for Flutamide and Flu-3 and 30–250 µg/mL for Flu-1 (for HPTLC), in the range of 2–50 µg/mL for both Flutamide and Flu-1, and in the range of 15–200 µg/mL for Flu-3 (for HPLC) were prepared in separate sets of 5 mL calibrated flasks. 0.5 mL urine was added to each concentration and the volume was adjusted by the appropriate solvent for each method. Samples were than filtered using 0.45 μm rated Acrodisc MS syringe filter (PN MS-3201). Instructions given under linearity for each method have been followed and calibration curves were then plotted.

#### Analysis of cytomed-250^®^ tablets

Samples equivalent to 1 µg/band and 15 µg/mL Flutamide were prepared from cytomed-250^®^ tablets solution and were analyzed by HPTLC and HPLC methods, respectively. Each sample was analyzed 5 times following the conditions illustrated under linearity of each method. The concentrations of the drug were calculated from the corresponding computed regression equations. To test the accuracy of the methods, standard addition technique was carried out by spiking the pre-analyzed cytomed-250^®^ samples with extra amounts of standard flutamide.

#### Statistical comparison

Data analysis was performed by comparing the results of each of the developed methods with those obtained by the reported BP [6] spectrophotometric method using student’s t and F tests.

## Results and discussion

Flutamide is an effective drug used in the treatment of prostatic carcinoma, it is rapidly metabolized in the body giving many metabolites including the toxic metabolite, Flu-1, which is one of the important metabolites in plasma, and Flu-3 which is the main urine inactive metabolite [4]. Lacking of analytical methods for determination of Flutamide and its metabolites inspired us for development of selective, sensitive, and accurate methods for quantitation of Flutamide, Flu-1, and Flu-3. The methods were extended for determination of the active drug and the studied metabolites in biological fluids including human plasma and urine. Nowadays, chromatographic methods became the analytical methods of choice for qualitative and quantitative pharmaceutical analysis [23–26].

In this work trials were done to develop HPTLC and HPLC methods which were able to separate and quantify the drug and its metabolites in short analysis time with high sensitivity and selectivity. Also, efforts were attempted to use less hazardous solvents. Organic solvents were classified into three categories according to their harmful environmental effects: desirable, acceptable, and undesirable [27]. Several trials were done to use desirable solvents, unfortunately all trials failed to separate all the studied components. Hence, acceptable solvents like cyclohexane, tetrahydrofurne, heptane, toluene (for HPTLC), and acetonitrile (for HPLC) were tried and the optimum ones were chosen. For the development of these analytical methods, Flu-1 and Flu-3 had to be synthesized in an adequate amount.

### Preparation of flutamide metabolites and structural elucidation

Synthesis of Flu-1 has been carried out following our method that was previously published [8]. Flu-3 preparation was carried out according to the synthetic pathway illustrated in Fig. 1.

Structural confirmation of the prepared metabolites has been performed by NMR analysis.

#### For Flu-1

**Fig. 2:**
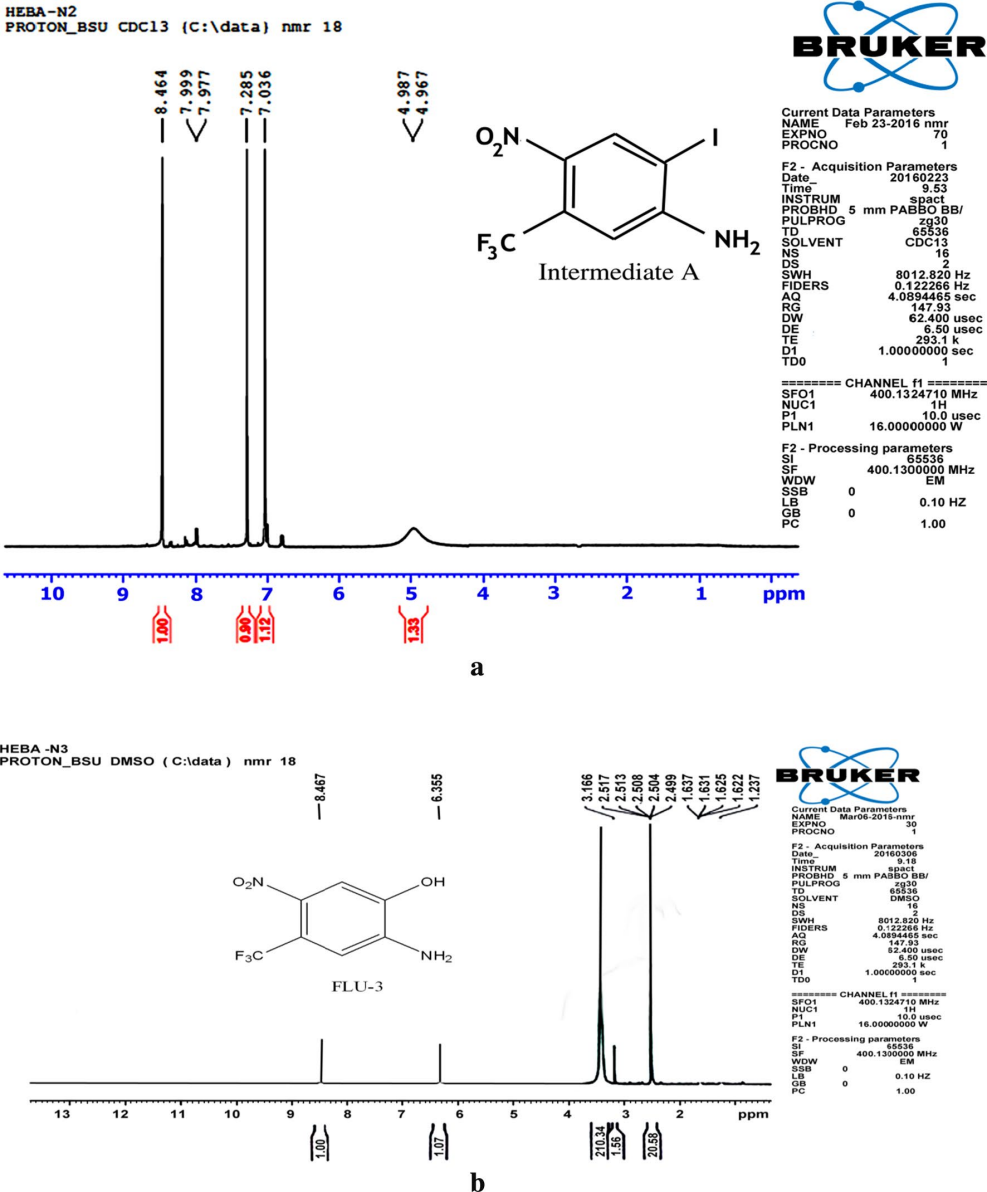
H-NMR of (**a**) intermediate (A) and **b** of Flu-3

The yield was 74%; it was a yellow powder; ^1^H NMR (CDCl_3_) δ 4.97 (br. s, 2H, NH_2_, D_2_O exchangeable), 7.03 (s, 1H, phenyl H-6), 8.46 (s, 1H, phenyl H-3) Fig. 2a.

#### For Flu-3

The yield was 82%; and it was a yellow powder; mp 197–199 °C; ^1^H NMR (DMSO-d6) δ 3.16 (br. s, 3H, NH_2_ and OH, D_2_O exchangeable), 6.35 (s, 1H, phenyl H-6), 8.46 (s, 1H, phenyl H-3) Fig. 2b.

### Method development and optimization

In order to achieve the chromatographic separation of the drug, its metabolites, and blind plasma or urine peaks and to improve symmetry of the peaks, various parameters such as the choice of mobile phase, its composition, and detection wavelength were considered during method optimization.

#### HPTLC method

Trials were made to choose a proper mobile phase to obtain maximum resolution and peak symmetry. Initially ethyl acetate together with several solvents including acetone, tetrahydrofurane, and toluene in different ratios were tried. All the trials gave bad resolution. Combination between tetrahydrofuran and toluene in different ratios were then tested, this resulted in slight improvement in chromatographic separation. In a trial to improve the separation between Flu-1 and Flu-3, mobile phase pH was changed by either using triethyl amine or glacial acetic acid. Using basic pH resulted in good separation but with tailed peak for Flu-3. Significant improvement was observed on using glacial acetic acid. Finally, the used mobile phase was toluene: tetrahydrofuran: glacial acetic acid (8:2:0.2, by volume). Saturation time did not significantly affect the method and so saturation time of 15 min was sufficient for good separation. Several scanning wavelengths were tested (220, 254, 300, and 370 nm). Detection at 220 nm resulted in high base line noise while 254 and 300 nm gave lower sensitivity. Detection at 370 nm was chosen that gave optimum signal to noise ratio for all the three components. In all trials plasma and urine peaks were almost retained on the stationary phase and did not interfere with the chromatographic separation.

**Fig. 3:**
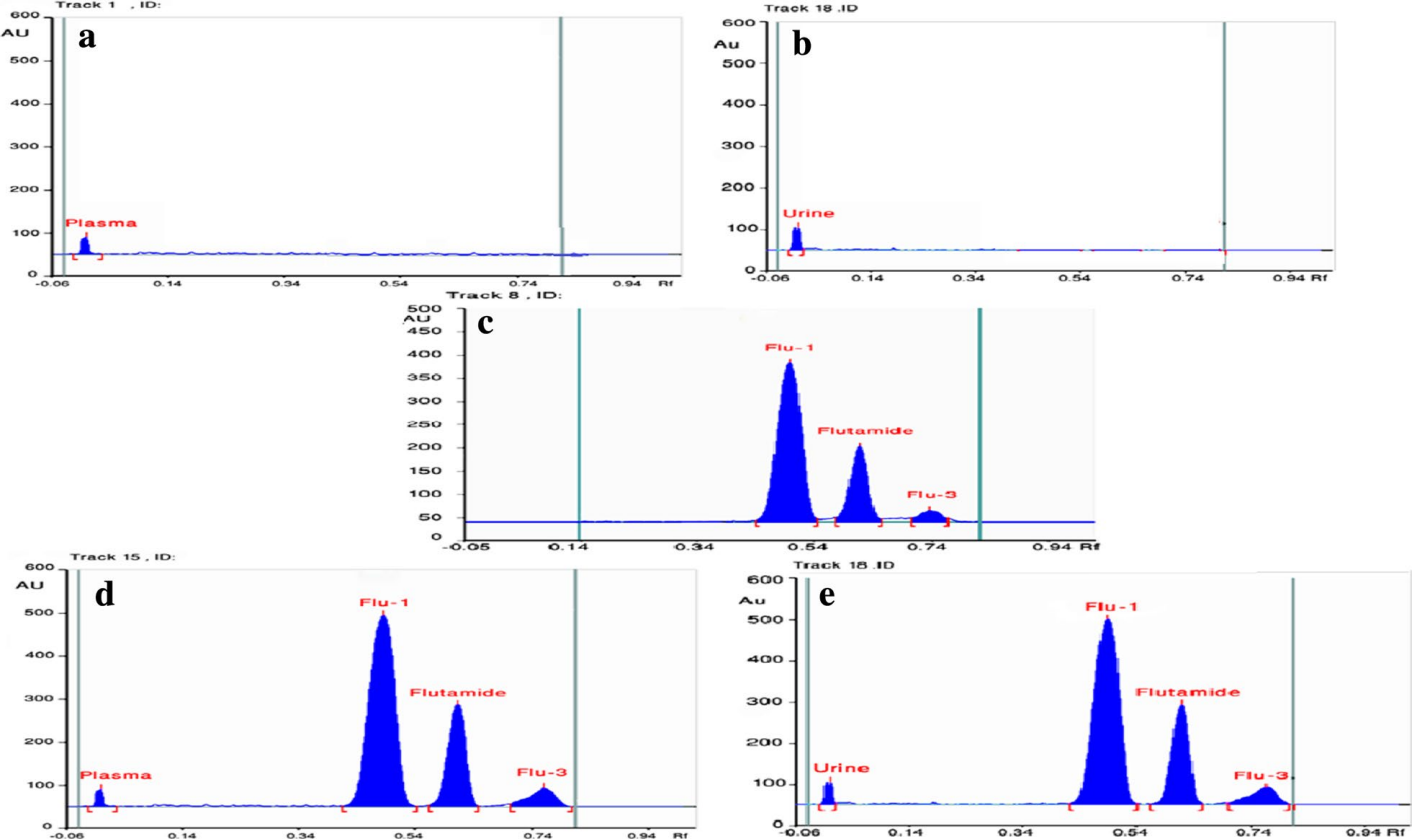
HPTLC chromatogram of a mixture of pure flutamide and its metabolites: **a** Blank plasma. **b** Blank urine. **c** Pure samples mixture. **d** Spiked human plasma mixture. **e** Spiked urine mixture

The optimum conditions for separation of the three studied components along with plasma or urine peaks were observed on using a mobile phase of toluene: tetrahydrofuran: glacial acetic acid (8:2:0.2, by volume), saturation time of 15 min and scanning at 370 nm, Fig. 3.

#### HPLC method

Initial trial was made following USP [7] reported HPLC method at which acetonitrile was the organic modifier and water was the aqueous solvent (45:55, v/v), flow rate = 1 mL/min with UV detection at 240 nm using C18 column as a stationary phase. Unfortunately, Flu-3 was highly retained (eluted after more than 15 min) and with very low sensitivity. Percentage of acetonitrile was then increased (up to 70%) but bad resolution was observed. Other trials were made by changing the mobile phase pH (3–9) using phosphoric acid, glacial acetic acid or triethyl amine, however, in vain. The stationary phase was then exchanged with C8 and CN columns. It was found that C8 gave the same results as C18 while CN column gave better results; Modification in the mobile phase strength was a must for complete resolution among Flu-1 and Flu-3. The ratio (40:60, v/v), acetonitrile: water gave complete resolution between the eluted peaks with appropriate analysis time. In order to increase sensitivity, different detection wavelengths were examined (220, 254, 300, and 370 nm). By observing UV spectra of the three components and after HPLC trials, one can conclude that wavelength 220 nm was suitable for detection of Flutamide, Flu-1, and Flu-3.

**Fig. 4:**
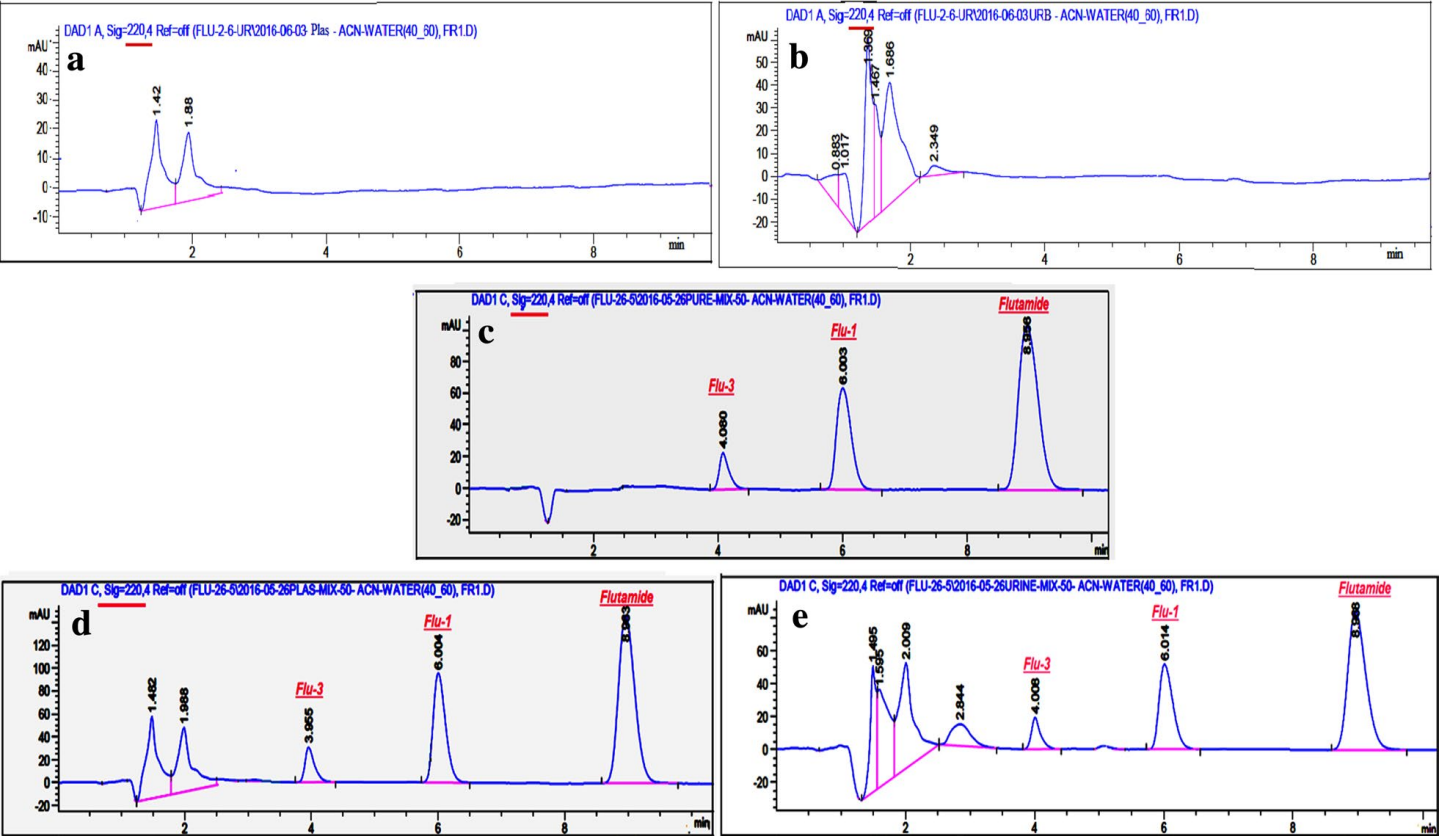
HPLC chromatogram of a mixture of flutamide, Flu-1 and Flu-3. **a** Blank plasma. **b** Blank urine. **c** Pure samples mixture. **d** Spiked human plasma mixture. **e** Spiked urine mixture

The studied components were completely resolved from each other and from either the plasma or urine peaks on using a CN column, mobile phase consisting of acetonitrile: water (40:60, v/v) with a flow rate of 1 mL/min and UV scanning at 220, Fig. 4.

### Method validation

#### Bio-analytical method validation

Instructions given by FDA [28] guidelines for Bio-analytical method validation was followed.

##### Linearity and limit of quantitation

On applying the developed methods to spiked human plasma and urine samples and then plotting the obtained peak areas of Flutamide, Flu-1, and Flu-3 against the corresponding concentrations, linear relations were obtained in different ranges and results are shown in Table 1. The lower limit of quantitation (LLOQ) was chosen according to FDA recommendations [28] at which LLOQ was accepted to be the lowest concentration on the calibration curve provided that the peak of the analyte was identifiable, reproducible, and had accuracy within 20% of the true concentration. LLOQ was 0.3 µg/b and for Flutamide, Flu-1, and Flu-3 in both spiked plasma and urine samples by HPTLC method, 2 µg/mL for Flutamide and Flu-1 and 15 µg/mL for FLu-3 in both spiked plasma and urine samples by HPLC method. The calculated value for each concentration was considered to be accepted when their deviation was ± 15% of the true ones except for LLOQ which was ± 20%.

**Table 1:** Assay and method validation parameters for the determination of flutamide and its metabolites in plasma and urine samples by the proposed methods

Parameters	HPTLC method						HPLC method					
	Spiked human plasma samples			Spiked urine samples			Spiked human plasma samples			Spiked urine samples		
	Flutamide	Flu-1	Flu-3	Flutamide	Flu-1	Flu-3	Flutamide	Flu-1	Flu-3	Flutamide	Flu-1	Flu-3
Range^a^	0.30–3.0	0.30–3.0	0.30–3.0	0.3–4.0	0.3–2.5	0.3–4.0	2–50	2–50	15–200	2–50	2–50	15–200
Slope	− 655.53^b^ 5414.98^c^	− 1659.60^c^ 12446.00^d^	− 455.09^c^ 2573.60^d^	− 261.41^c^ 41861.50^d^	− 2182.80^c^ 13125.00^d^	− 139.66^c^ 1829.70^d^	60.0650	41.5830	5.8755	51.8810	37.2350	6.3446
Intercept	665.50	3039.00	1240.80	1385.10	3066.40	305.60	− 13.2490	− 2.8848	25.9910	− 21.3530	− 0.8850	35.0270
Correlation (r)	0.9998	0.9998	0.9992	0.9997	0.9994	0.9996	0.9999	0.9997	0.9999	0.9998	0.9999	0.9997
LLOQ	0.30	0.30	0.30	0.30	0.30	0.30	2.0	2.0	15.0	2.0	2.0	15.0

^a^Range: for HPTLC, it is measured by µg/band and for HPLC method in µg/mL

The linearity was achieved using the polynomial regression equation: A = aX^2^ + bX + C

^b^Coefficient 1, ^c^Coefficient 2X = concentration µg/band, C = intercept

##### Selectivity

Chromatograms in Figs. 3 and 4 showed that there was no interference from endogenous components in plasma and urine matrices and no additional interfering peaks were observed. Blank plasma and urine samples were obtained from six healthy volunteers.

##### Precision and accuracy

Repeatability and intermediate precision expressed as relative standard deviation (RSD) were tested by analyzing four different samples, 5 times each (including LLOQ and other three quality control samples). All results in Table 2 did not exceed the acceptance criteria which were ≤ 15% (for quality control samples) and 20% for LLOQ. Additionally, accuracy was tested by the same way as precision and was calculated as percentage recovery. The mean values of each component in each of the developed methods did not exceed ± 15% (for quality control samples) and 20% (for LLOQ), Table 2.

**Table 2:** Intra and inter assay precision and accuracy

Component	Concentration (µg/band)^a^	Intraday			Interday		
		Recovery %	Bias %^b^	RSD%	Recovery %	Bias%	RSD%
a. For HPTLC method							
In plasma							
Flu	0.3 (LLOQ)	93.98	− 6.02	6.526	93.97	− 6.03	8.169
	0.5 (LQC)	97.88	− 2.12	3.183	98.79	− 1.21	4.33
	1.6 (MQC)	103.54	3.54	3.063	105.353	5.53	4.538
	2.5 (HQC)	98.61	− 1.39	2.42	104.63	4.63	5.97
Flu-1	0.3 (LLOQ)	103.69	3.69	6.191	106.49	6.49	7.954
	0.5 (LQC)	103.61	33.61	3.703	104.95	4.95	4.085
	1.6 (MQC)	98.11	− 1.89	4	97.99	− 2.01	4.614
	2.5 (HQC)	97.87	− 2.13	3.053	96.84	− 3.16	4.917
Flu-3	0.3 (LLOQ)	105.34	5.34	7.934	96.19	− 3.81	9.088
	0.5 (LQC)	102.36	2.36	4.129	104.76	4.76	5.688
	1.6 (MQC)	102.93	2.93	4.61	104.38	4.338	5.699
	2.5 (HQC)	97.338	− 2.62	4.645	98.61	1.39	6.788
In urine							
Flu	0.3 (LLOQ)	101.13	1.13	4.978	104.84	4.84	8.015
	0.5 (LQC)	95.78	− 4.22	3.193	101.73	1.73	7.778
	1.6 (MQC)	98.75	− 1.25	2.051	99.82	− 0.18	2.797
	3 (HQC)	98.31	− 1.69	2.446	98.99	− 1.01	3.597
Flu-1	0.3 (LLOQ)	97.14	− 2.86	7.44	107.87	7.87	7.694
	0.5 (LQC)	98.93	− 1.07	3.282	98.2	− 1.8	4.157
	1.6 (MQC)	103.29	3.29	2.679	98.06	− 1.94	2.985
	2 (HQC)	99.23	− 0.77	2.771	97.89	− 2.11	4.175
Flu-3	0.3 (LLOQ)	103.82	3.82	8.481	106.31	6.31	9.175
	0.5 (LQC)	102.7	2.7	3.223	104.1	4.1	3.801
	1.6 (MQC)	97.82	− 2.18	2.685	101.96	1.96	3.698
	3 (HQC)	98.67	− 1.33	2.428	97.94	− 2.06	5.032

Component	Concentration (µg/band)^a^	Intraday			Interday		
		Recovery %	Bias %^b^	RSD%	Recovery %	Bias %	RSD%
b. For HPLC method							
In plasma							
Flu	2 (LLOQ)	102.04	2.04	4.389	109.41	9.41	8.903
	5 (LQC)	97.98	− 2.02	3.368	102.63	2.63	5.329
	20 (MQC)	98.97	− 1.03	2.319	97.82	− 2.18	3.572
	45 (HQC)	98.56	− 1.44	3.404	99.28	− 0.72	4.138
Flu-1	2 (LLOQ)	96	− 4	5.657	104.32	4.32	10.386
	5 (LQC)	98.29	− 1.71	3.282	98.57	− 1.43	5.77
	20 (MQC)	98.98	− 1.02	1.982	99.83	− 0.17	2.317
	45 (HQC)	97.52	− 2.48	2.271	98.86	− 1.14	2.321
Flu-3	15 (LLOQ)	100.98	0.98	3.894	104.4	4.4	6.945
	30 (LQC)	98.6	− 1.4	3.075	101.44	1.44	5.8
	100 (MQC)	100.54	0.54	3.494	103.25	3.25	6.2
	170 (HQC)	98.54	− 1.46	3.92	95.79	− 4.21	6.116
In urine							
Flu	2 (LLOQ)	101.91	1.91	4.298	98.84	− 1.16	7.831
	5 (LQC)	97.71	− 2.29	4.01	97.27	− 2.73	4.968
	20 (MQC)	103.16	3.16	3.415	101.29	1.29	6.772
	45 (HQC)	101.59	1.59	3.353	104.4	4.4	4.92
Flu-1	2 (LLOQ)	98.82	− 1.18	3.734	96.8	− 3.2	8.483
	5 (LQC)	99.57	− 0.43	2.051	97.99	− 2.01	3.594
	20 (MQC)	99.29	− 0.71	2.144	104.29	4.29	4.705
	45 (HQC)	101.19	1.19	1.652	105.15	5.15	4.46
Flu-3	15 (LLOQ)	102.31	2.31	5.014	103.94	3.94	5.014
	30 (LQC)	99.17	− 0.83	2.228	102.31	2.31	4.49
	100(MQC)	100.55	0.55	1.79	102.45	2.45	3.994
	170 (HQC)	100.32	0.32	1.335	101.37	1.37	2.533

^a^Average of 5 experiments

^b^% of deviation from true value

##### Recovery

It was calculated as % recovery and obtained by comparing the peak areas of analytes in plasma (after removal of plasma protein) with those of pure samples of the same concentrations. Recovery was performed at three concentration levels (low, medium, and high). The recovery ranged from 94.56 to 97.96%, 94.53 to 96.94% and 92.02 to 98.18% for Flutamide, Flue 1, and Flu-3, respectively (for HPTLC method). While for HPLC, it was in the range of 94.87–99.47%, 94.78–98.83%, and 93.50–96.91%, respectively.

##### Sample stability

###### Freeze and thaw cycle

To test samples stability in both plasma and urine, human plasma and urine were spiked with definite concentrations of Flutamide, Flu-1, and Flu-3. Samples were stored at − 20 °C and subjected to three freeze–thaw cycles. The recovery percentages were calculated for each concentration for which the corresponding standard deviations (SD) were calculated. Sample stability was confirmed when a change of less than 15% of the analyte concentration was observed [29]. Satisfactory results were obtained, verifying no significant loss of the analytes concentrations during the repeated freezing and thawing as shown in Table 3.

**Table 3:** Results of freezing–thawing and short term stability study

	Spiked human plasma			Spiked urine		
	Flutamide	Flu-1	Flu-3	Flutamide	Flu-1	Flu-3
Freezing thaw cycle						
HPTLC method						
SD^a^	3.143	1.725	2.901	2.524	1.301	3.083
HPLC method						
SD^a^	2.028	1.75	1.730	2.21	2.402	1.980
Short term stability						
HPTLC method						
SD^a^	2.44	2.86	3.18	2.60	3.01	3.08
HPLC method						
SD^a^	2.00	1.54	3.04	2.43	2.64	3.52

^a^Average of 3 determinations

###### Short term temperature stability

Analysis of quality control samples left for 24 h at room temperature was carried out and results are shown in Table 3 which proved stability of all samples under working conditions.

#### Analytical method validation

USP [7] instructions for method validation have been followed during method validation step.

Linearity, accuracy, precision, LOD and LOQ were evaluated and the results are summarized in Table 4.

**Table 4:** Assay and method validation parameters for the determination of flutamide and its metabolites in pure samples by the proposed methods

Parameters	HPTLC method			HPLC method		
	Pure samples			Pure samples		
	Flutamide	Flu-1	Flu-3	Flutamide	Flu-1	Flu-3
Range^a^	0.1–3	0.3–2.5	0.3–3.5	2–50	1–50	5–200
Slope	− 645.07^b^ 4902.40^c^	− 2070.03^c^ 13121^d^	− 101.45^c^ 825.60^d^	43.6020	34.9780	6.4161
Intercept	464.92	2938.80	1275.60	− 17.9250	− 5.1212	5.4658
Correlation (r)	0.9998	0.9998	0.9999	0.9998	0.9999	0.9998
Accuracy Precision (SD)^d^	99.98	100.99	100.67	99.86	99.45	99.57
Repeatability^e, f^	1.12	0.35	2.47	0.863	1.224	1.016
Intermediateprecision^e, g^	1.59	1.37	2.86	1.144	1.334	1.027
LOD^h^	0.03	0.09	0.09	0.45	0.31	1.65
LOQ^i^	0.09	0.28	0.27	1.35	0.93	4.95

^a^Range: for HPTLC, it is measured by µg/band and for HPLC method in µg/mL

The linearity was achieved using the polynomial regression equation: A = aX^2^ + bX + C

^b^Coefficient 1, ^c^ Coefficient 2 X = concentration µg/band C = intercept

^d^Accuracy: ^a^ Mean of 9 concentrations of each component

^e^Average of three experiments

^f^Standard deviation of 3 concentrations of each component (0.5, 1.5 and 2 µg/band) for HPTLC method and 10, 20 and 30 µg/band (for flutamide and Flu-1), 50, 100 and 150 µg/mL (for Flu-3) for HPLC method on the same day

^g^Standard deviation of 3 concentrations of each component (0.5, 1.5 and 2 µg/band) for HPTLC method and 10, 20 and 30 µg/band (for flutamide and Flu-1), 50, 100 and 150 µg/mL (for Flu-3) for HPLC method on three successive days

^h^LOD = (3.3 X SD)/slope (SD of the intercept using the lower part of the calibration graph, the slope of the calibration curve)

^i^LOQ = (10X SD)/slope (SD of the intercept using the lower part of the calibration graph, the slope of the calibration curve)

##### Selectivity of the method

Was proved by the complete separation of the drug and the metabolites under the applied chromatographic conditions, Figs. 3 and 4. Specificity was also examined by analyzing the commercial tablets, results in Table 5 proved that excipients did not interfere.

**Table 5:** Determination of Flutamide in its pharmaceutical formulation by the proposed methods, application of standard addition technique

Pharmaceutical formulation	HPTLC method				HPLC method			
	Taken (µg/band)	Found^a^ % ± % RSD	Added (µg/band)	Recovery^b^ %	Taken (µg/mL)	Found^a^ % ± %RSD	Added (µg/mL)	Recovery^b^ %
Cytomed^®^ tablets labeled to contain 250 mg flutamide/tablet	1.00	101.75 ± 0.975	0.60	100.63	15.00	102.02 ± 1.002	10.00	100.90
			1.00	97.00			12.00	100.47
			1.50	99.0			15.00	98.13
	Mean ± SD			98.82 ± 1.818	Mean ± SD			99.52 ± 1.385

^a^Average of 5 determinations

^b^Average of 3 determinations

##### Robustness

Was studied and all the obtained values were < 3 indicating that the proposed methods were not affected by the small variations made in the studied parameters, Table 6.

**Table 6:** Robustness and ruggedness studies of the developed method

HPTLC method			
Robustness (SD)^a^			
Factor	Flu-1	Flutamide	Flu-3
1-Amount of acetic acid (± 0.01 mL/min)	0.03	0.03	0.03
2-% Tetrahydrofuran in the mobile phase (± 1%)	0.62	1.25	0.94
3-Detection wavelength (± 2 nm)	1.1	0.73	0.86
Ruggedness (SD)^a^			
1-Two analysts	0.009	0.004	0.009

HPLC method			
Robustness (SD)^a^			
Factor	Flutamide	Flu-1	Flu-3
1-Mobile phase flow rate (± 0.05 mL/min)	2.916	0.742	0.446
2-% acetonitrile in the mobile phase (± 1%)	2.423	2.604	2.964
3-Detection wavelength (± 2 nm)	0.456	0.582	0.516
Ruggedness (SD)^a^			
1-Two analysts	0.196	0.412	0.269
2-Different acetonitrile manufacturer	0.939	1.33	0.287

^a^Average of 3 determinations

### System suitability testing parameters

System suitability was performed by calculating different chromatographic parameters. Results presented in Table 7 showed that the values of selectivity and resolution factors are within the accepted limits [30] indicating good chromatographic separation.

**Table 7:** System suitability testing parameters of the developed methods

Parameters	HPTLC method			HPLC method		
	Flu-1	Flutamide	Flu-3	Flu-3	Flu-1	Flutamide
R_f_ (for HPTLC) or Rt (for HPLC)	0.48 ± 0.01	0.6 ± 0.01	0.74 ± 0.02	4.01 ± 0.06	6.00 ± 0.01	8.96 ± 0.02
Peak symmetry	1.00	1.00	0.94	1.30	1.25	1.10
Selectivity (α)						
*Plasma*	9.14	1.23	1.24	3.54	1.71	1.62
*Urine*	9.8			1.71		
Resolution (R_s_)						
*Plasma*	10.18	2.00	2.24	4.60	5.24	5.60
*Urine*	9.58			2.77		
Capacity factor (α)	1.04	0.64	0.32	2.34	4.00	6.48
Number of theoretical plates (N)				3059.22	2515.28	3698.29
Height equivalent to theoretical plate (H) (in cm)				0.0049	0.0060	0.0041

### Application of the method

After optimization and validation of the methods, they were further tested by application to cytomed-250^®^ tablets, the % recoveries were found to be 101.75 ± 0.975 and 102.02 ± 1.002 for HPTLC and HPLC methods, respectively indicating that tablets common excipients did not interfere. Standard addition technique has been carried out to further access accuracy of the methods where the obtained results, Table 5, proved the accuracy of the proposed methods.

### Statistical comparison

One-way analysis of variance (ANOVA) is applied to test the significant difference between the means of three or more unrelated groups. This test was used here to compare the results obtained by applying the suggested methods to available pharmaceutical formulation and those gained by applying the official method [6]. The results showed that the value of F_(calculated)_ [3.069] was lower than F_(critical)_ [3.885] and p value = 0.084 indicating no significant difference between the three methods. Additionally, student’s t test was used to test the significance among each of the developed methods and the official one [6]. The calculated t value was found to be 1.847 and 2.216 for each of the HPTLC and HPLC methods, respectively while the tabulated t at p = 0.05 was 2.306 which meant that there was no significant difference between each of the two methods and the official one with 95% confidence limit. The developed methods had advantages over the official one of being more selective and able to resolve the drug even in presence of plasma and urine matrices. In addition, chromatographic methods are known to be of higher sensitivity than spectrophotometric methods, hence the developed methods were used to quantify the drug along with its metabolites.

In the same way, the developed HPLC method was compared with all the published HPLC methods [3, 7, 16–20] regarding the used chromatographic conditions and the resulted retention time. Comparison items and results are given in Table 8. The results of this comparison showed that the method is the unique one that determined the drug and the metabolites in single run within short analysis time. Moreover, it is the only one that was applied to pharmaceutical formulation, spiked human plasma, and urine. Additionally, the developed HPTLC method is the first developed one for analysis of Flutamide.

**Table 8:** Comparison between the developed and the published HPLC methods

The method	The chromatographic condition
The developed HPLC method	*Stationary phase*: CN column *Mobile phase:* acetonitrile: water (40:60, v/v) *Flow rate:* 1 mL/min *Retention time:* 8.96 min *Linearity range:* 2–50 µg/mL *Detection wavelength:* 220 nm
Determination of flutamide in tablets [3]	*Stationary phase:* Luna C18 column *Mobile phase:* 0.05 M phosphate buffer (pH = 4): acetonitrile (50:50, v/v) *Flow rate:* 1 mL/min *Retention time:* 5.57 min *Linearity range:* 2.9–11.6 µg/mL
HPLC method for flutamide in pure form and dosage form [7]	*Stationary phase:* Packing L1 column *Mobile phase:* Acetonitrile: water (55:45, v/v). *Flow rate:* 1 mL/min *Detection wavelength:* 240 nm *Retention time:* 1 min
Stability study of flutamide [16]	*Stationary phase:* Licrospher RP-18. *Mobile phase:* Acetonitrile: water: methanol (30:45:25, by volume). *Flow rate:* 1 mL/min *Detection wavelength:* 299 nm
Stability study of flutamide [17]	*Stationary phase:* C18 column *Mobile phase:* methanol: 0.04 M phosphate buffer (pH = 4) (75:25, v/v). *Detection wavelength:* 240 nm *Retention time:* 4 min *Linearity range*: 0.2–25 µg/mL
HPLC determination of flutamide [18]	*Stationary phase:* nucleosil C18 column *Mobile phase:* acetonitrile: water (60:40, v/v). *Flow rate:* 1 mL/min *Retention time:* 6.35 min *Linearity range:* 0.0125–0.625 µg/mL
Determination of flutamide in pharmaceutical formulation [19]	*Stationary phase:* Spheri-5 RP-18 column *Mobile phase:* acetonitrile: water (70: 30, v/v) *Flow rate:* 1 mL/min *Detection wavelength:* λ_ex_ = 255 nm, λ_em_ = 375 nm *Retention time:* 3.9 min *Linearity range:* 0.1–0.6 µg/mL
HPLC determination of flutamide [20]	*Stationary phase:* C8 column *Mobile phase:* methanol: acetonitrile: KH2PO4 (50 mM, pH = 3.2) (29:38:33, by volume) *Flow rate:* 1 mL/min *Retention time:* 2.9 min *Linearity range:* 0.0625–1.6 µg/mL

## Conclusion

The developed HPTLC and HPLC–DAD methods are accurate, precise, selective, and sensitive. Validation parameters prove that the methods are suitable for the analysis of Flutamide as bulk drug, in pharmaceutical formulation, and in the presence of drug metabolites, Flu-1 and Flu-3. The methods have been successfully applied for different biological fluids including urine and plasma samples. Comparing the developed methods with the official BP spectrophotometric method showed that they were more selective, sensitive, and had the advantages of simultaneous quantitation of Flutamide and its metabolites in a single run and scanning wavelength.

## References

[1] Budavari S (2003). The merck index.

[2] Sternal R, Nugara N (2001). Analytical Profiles of Drug Substances and Excipients.

[3] Salgado HRN, Menezes M, Storti MPB (2005). Determination of flutamide in tablets by high-performance liquid chromatography. Acta Farmaceutica Bonaerense.

[4] Tevell A (2006). Flutamide metabolism in four different species in vitro and identification of flutamide metabolites in human patient urine by high performance liquid chromatography/tandem mass spectrometry. Drug Metabol Dispos.

[5] Goda R (2006). Detection of a new *n*-oxidized metabolite of flutamide, *n*-[4-nitro-3- (trifluoromethyl)phenyl]hydroxylamine, in human liver microsomes and urine of prostate cancer patients. Drug metabol. Dispos.

[6] The British Pharmacopoeia (2007). Her Majesty’s.

[7] The United States Pharmacopeia (2012). National Formulary 35.

[8] Farid NF, Abdelwahab NS (2015). Two different spectrophotometric determinations of potential anticancer drug and its toxic metabolite. Spectrochim Acta.

[9] Ensafi AA, Khoddami E, Rezaei B (2016). Development of a cleanup and electrochemical determination of flutamide using silica thin film pencil graphite electrode functionalized with thiol groups. J Iranian Chem Soc.

[10] Temerk YM, Ibrahim HSM, Schuhmann W (2016). Square wave cathodic adsorptive stripping voltammetric determination of the anticancer drugs flutamide and irinotecan in biological fluids using renewable pencil graphite electrodes. Electroanalysis.

[11] Reddy MN, Murthy TK, Reddy MD, Sankar DG (2001). New spectrophotometric methods for determination of flutamide. Asian J Chem.

[12] Reddy KM, Suvardhan K, Suresh K, Prabahar S, Chiranjeevi P (2003) Proceedings of the third International Conference on Environmental and Health, Chennai, India, 5–17 December. Chennai: Department of Geogaphy, University of Madras and Faculty of Environmental Studies, York University, 410–416

[13] Reddy MN, Murthy TK, Reddy MD, Sankar DG (2001). Spectrophotometric estimation of flutamide in pharmaceutical dosage forms. Asian J Chem.

[14] Trazanavaras PD, Themelis DG (2007). Automated determination of flutamide by a validated flow injection method: application to dissolution studies of pharmaceutical tablets. J Pharm Biomed Anal.

[15] Smith AA, Manavalan R, Kannan K, Rajendiran N (2008). Spectrofluorimetric determination of flutamide in pharmaceutical preparations. Orien J Chem.

[16] Miranda A, Caramballo I, Millaacuten M (2002). Stability study of flutamide in solid state and in aqueous solution. Drug Dev Indust Pharm.

[17] El-Shaheny RN, Yamadaa AB, Yamadaa K (2015). The influence of pH and temperature on the stability of flutamide. An HPLC investigation and identification of the degradation product by EI^+^-MS. RSC Adv.

[18] Filip M, Coman V, Avram V, Coman I (2007) Proceeding in the 1st international conference on advancements of medicine and health care through technology. Romania: Medi-Tech

[19] Smith AA, Manavalan R, Kannan K, Rajendrin N (2009). Improved liquid chromatographic method for the determination of flutamide in pharmaceutical formulation. Int J Pharm Technol Res.

[20] Esmaeilzadeh S, Valizadeh H, Zakeri-Milani P (2016). A simple, fast, low cost, HPLC/UV validated method for determination of flutamide: application to protein binding studies. Adv Pharm Bull.

[21] Capello Ch, Fischer U, Hungerbühler K (2007). What is a green solvent? A comprehensive framework for the environmental assessment of solvents. Green Chem.

[22] Jessop PG (2011). Searching for green solvents. Green Chem.

[23] Abdelaleem EA, Abdelwahab NS (2013). Validated stability indicating RP-HPLC method for determination of paracetamol, methocarbamol and their related substances. Anal Methods.

[24] Farid NF, Abdelaleem EA (2016). HPTLC method for the determination of paracetamol, pseudoephedrine and loratiding in tablets and human plasma. J Chromatogr Sci.

[25] Abdelwahab NS, Farid NF (2014). Validated HPLC–DAD method for stability study of sulbutiamine HCl. RSC Adv.

[26] Abdelwahab NS, Abdelrahman MM (2015). Stability indicating RP-HPLC method for the determination of flubendazole in pharmaceutical dosage forms. RSC Adv.

[27] Palos-Pacheco R (2013) Solvents in the environment. Greening research education and environment network. https://greeningresearch.com. Access 30 Oct 2017

[28] FDA (2001) Guidance for industry: bioanalytical method validation. US Department of Health and Human Services. Food and Drug Administration, Center for Drug Evaluation and Research (CDER), Center for Veterinary Medicine (CV)

[29] Rezk MR, Basalious EB, Karim IA (2015) Development and validation of sensitive and rapid UPLC-MS/MS method for quantitative determination of daclatasvir in human plasma: Application to a bioequivalence study. J Pharm Biomed Anal http://dx.doi.org/10.1016/j.jpba.2015.05.006 10.1016/j.jpba.2016.05.016 27232152

[30] Fried B, Sherma J (1999). Thin layer chromatography.

